# Supplementary material for: Empirical methods for controlling false positives and estimating confidence in ChIP-Seq peaks
Source: BMC Bioinformatics. 2008 Dec 5;9:523. doi: 10.1186/1471-2105-9-523 (PMC2628906; doi:10.1186/1471-2105-9-523)
Supplement: Additional File 1 — A variety of html documents from the USeq web site detailing the available applications, their best usage, output file type descriptions, command line menus, etc. [file 1471-2105-9-523-S1.zip › USeqUserGuides/JavaDoc.zip!/JavaDoc/edu/utah/seq/parsers/ElandMultiParser.html]

ElandMultiParser


|  |  |  |
| --- | --- | --- |
| **Overview** \| **Package** \| **Class** \| **Use** \| **Tree** \| **Deprecated** \| **Index** \| **Help** | |  |
| **PREV CLASS**   **NEXT CLASS** | **FRAMES**    **NO FRAMES**     **All Classes** |  |
| SUMMARY: NESTED \| FIELD \| CONSTR \| METHOD | DETAIL: FIELD \| CONSTR \| METHOD |  |


---


## edu.utah.seq.parsers Class ElandMultiParser

```
java.lang.Object
  edu.utah.seq.parsers.ElandMultiParser
```

---

``` public class ElandMultiParser extends java.lang.Object ```

Parses an Eland xxx.eland\_multi.txt file tabulating hits to each fasta entry. Good for scoring hits to a transcriptome where
every fasta entry represents a different gene.

**Author:**
:   david.nix@hci.utah.edu

---

**Constructor Summary**

`ElandMultiParser(java.lang.String[] args)`


| **Method Summary** | |
| --- | --- |
| `static java.lang.String` | `formatFraction(int number, int total, int numberDecimals)`             Returns number space (percent%) |
| `void` | `loadHits(java.lang.String alignments)` |
| `static void` | `main(java.lang.String[] args)` |
| `void` | `parseAndLoad(java.io.File multiFile)` |
| `static void` | `printDocs()` |
| `void` | `printOutMap()` |
| `void` | `processArgs(java.lang.String[] args)`             This method will process each argument and assign new varibles |

**Methods inherited from class java.lang.Object**

`equals, getClass, hashCode, notify, notifyAll, toString, wait, wait, wait`

**Constructor Detail**

### ElandMultiParser

```
public ElandMultiParser(java.lang.String[] args)
```


**Method Detail**

### printOutMap

```
public void printOutMap()
```

---


### parseAndLoad

```
public void parseAndLoad(java.io.File multiFile)
```

---


### loadHits

```
public void loadHits(java.lang.String alignments)
```

---


### formatFraction

```
public static java.lang.String formatFraction(int number,
                                              int total,
                                              int numberDecimals)
```

:   Returns number space (percent%)

---


### main

```
public static void main(java.lang.String[] args)
```

---


### processArgs

```
public void processArgs(java.lang.String[] args)
```

:   This method will process each argument and assign new varibles

---


### printDocs

```
public static void printDocs()
```


---


|  |  |  |
| --- | --- | --- |
| **Overview** \| **Package** \| **Class** \| **Use** \| **Tree** \| **Deprecated** \| **Index** \| **Help** | |  |
| **PREV CLASS**   **NEXT CLASS** | **FRAMES**    **NO FRAMES**     **All Classes** |  |
| SUMMARY: NESTED \| FIELD \| CONSTR \| METHOD | DETAIL: FIELD \| CONSTR \| METHOD |  |


---
